# Association between the plasma/whole blood lead ratio and history of spontaneous abortion: a nested cross-sectional study

**DOI:** 10.1186/1471-2393-7-22

**Published:** 2007-09-27

**Authors:** Héctor Lamadrid-Figueroa, Martha M Téllez-Rojo, Mauricio Hernández-Avila, Belem Trejo-Valdivia, Maritsa Solano-González, Adriana Mercado-Garcia, Donald Smith, Howard Hu, Robert O Wright

**Affiliations:** 1 Division of Program Evaluation and Biostatistics, Center for Research in Evaluation and Surveys, National Institute of Public Health, Cuernavaca, México; 2 Ministry of Health, México City, Mexico; 3 Center for Research in Mathematics (CIMAT), Aguascalientes, México; 4 Division of Environmental Health, Center for Population Health Research, National Institute of Public Health, Cuernavaca, México; 5 Environmental Toxicology, University of California at Santa Cruz, Santa Cruz CA, USA; 6 Department of Environmental Health Sciences, University of Michigan School of Public Health, Ann Arbor MI, USA; 7 Department of Environmental Health, Harvard School of Public Health, Boston MA, USA

## Abstract

**Background:**

Blood lead has been associated with an elevated risk of miscarriage. The plasmatic fraction of lead represents the toxicologically active fraction of lead. Women with a tendency to have a higher plasma/whole blood Pb ratio could tend towards an elevated risk of miscarriage due to a higher plasma Pb for a given whole blood Pb and would consequently have a history of spontaneous abortion.

**Methods:**

We studied 207 pregnant Mexico City residents during the 1^st ^trimester of pregnancy, originally recruited for two cohorts between 1997 and 2004. Criteria for inclusion in this study were having had at least one previous pregnancy, and having valid plasma and blood Pb measurements. Pb was measured in whole blood and plasma by inductively coupled plasma mass spectrometry using ultra-clean techniques. History of miscarriage in previous pregnancies was obtained by interview. The incidence rate of spontaneous abortion was defined as the proportion of previous pregnancies that resulted in miscarriage. Data were analyzed by means of Poisson regression models featuring the incidence rate of spontaneous abortion as the outcome and continuous or categorized plasma/blood Pb ratios as predictor variables. All models were adjusted for age and schooling. Additionally, logistic regression models featuring inclusion in the study sample as the outcome were fitted to assess potential selection bias.

**Results:**

The mean number of miscarriages was 0.42 (range 0 to 4); mean Pb concentrations were 62.4 and 0.14 μg/L in whole blood and plasma respectively. Mean plasma/blood Pb ratio was 0.22%. We estimated that a 0.1% increment in the plasma/blood Pb ratio lead was associated to a 12% greater incidence of spontaneous abortion (p = 0.02). Women in the upper tertile of the plasma/blood Pb ratio had twice the incidence rate of those in the lower tertile (p = 0.02). Conditional on recruitment cohort, inclusion in the study sample was unrelated to observable characteristics such as number of abortions, number of pregnancies, blood Pb levels, age schooling, weight and height.

**Conclusion:**

Women with a large plasma/whole blood Pb ratio may be at higher risk of miscarriage, which could be due to a greater availability of placental barrier-crossing Pb.

## Background

Several studies have reported a positive association between maternal blood lead concentration and the risk of spontaneous abortion [1-3]. This is of concern since one of these studies showed an important increase in the risk of miscarriage even among women with low to moderate blood lead levels [1]. However, previous studies were not conclusive, which could be attributed to methodological problems in study designs [4].

An additional explanation for the inconclusive results is that blood Pb may not be the optimal biomarker to assess lead exposure. Over 99 percent of blood Pb is bound to erythrocytes, which cannot cross the placental barrier, thus, plasma lead concentration has been suggested as a better surrogate for the toxicologically active fraction of lead in blood [5,6]. This has been substantiated by the findings that plasma Pb was a greater predictor of toxicity on hematopoiesis than blood Pb [7], and of delays in neurobehavioral development after fetal exposure to Pb [8]. To our knowledge, only one study assessed whether plasma lead concentrations where associated to the occurrence of miscarriage, and although the authors could not find an association [9], their study had a rather small sample size (n = 40), and plasma lead has a large inter-individual variability [10,11]. The latter could make it difficult to find associations if the time between the exposure measurement and the event is relatively large.

To date, there have been no studies relating the plasma/whole blood Pb ratio, with adverse health outcomes. It has been previously shown that the plasma whole/blood Pb ratio is highly influenced by interindividual factors [12]. Such factors could be polymorphic alleles of genes coding for proteins involved in the partitioning of circulating lead, the most important being δ-aminolevulinic acid dehydratase (ALAD), among other unidentified binding sites for lead [13,14]. A recent contribution described that polymorphisms in the ALAD genes are strongly associated to plasma/blood Pb ratios [15]. This finding raises the possibility that fetuses of women with a tendency to have a lower erithrocytic lead binding capacity, reflected in higher plasma/blood Pb ratios, and consequently greater plasma Pb levels for a given whole blood Pb concentration, would be more exposed to lead and at a greater risk of reproductive toxicity [11]; under this hypothesis, current blood or plasma Pb would not necessarily be strongly associated to history of miscarriage, since blood or plasma Pb concentrations are more dependent on intra-individual temporal variation than plasma/blood Pb ratios and would therefore be less correlated between pregnancies. Also, if this hypothesis is true then the plasma/blood Pb ratio could be viewed as a marker of susceptibility for the toxic effects of Pb. To test this hypothesis, we examined the association between the plasma/blood Pb ratio and the past history of miscarriages among two cohorts of pregnant women residing in Mexico City.

## Methods

### Study population

**Figure 1:**
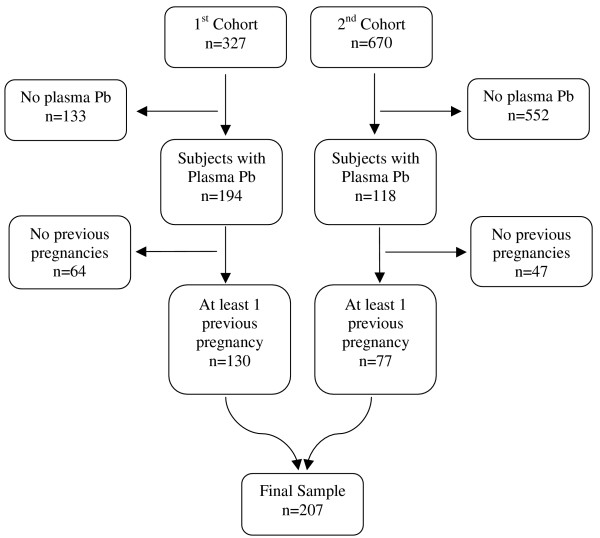
**Composition of the study sample. Mexico City, Mexico, 1997–2004**. Composition of the sample in a study to evaluate the association between plasma/blood Pb ratios and history of spontaneous abortion.

We conducted a nested cross-sectional study on 207 healthy pregnant women, recruited to participate in two larger cohort studies. The first cohort was recruited from May 1997 through July 1999, and the 2^nd ^from January 2001, to April 2004. The original objectives of the first cohort were to assess lead toxicokinetics and bone metabolism during pregnancy, as well as investigating a possible link between prenatal exposure to lead and neurodevelopment later in life. The second cohort was a randomized clinical trial to evaluate the efficacy of a calcium supplementation during pregnancy on diminishing blood Pb concentration. Inclusion & exclusion criteria for both cohorts were the same: participants were selected from healthy pregnant women with a maximum of 14 weeks of gestation with a confirmed positive beta-HCG test, who were invited to participate during their prenatal care visits to one of three clinics of the Mexican Institute of Social Security (IMSS) in the Mexico City area. Exclusion criteria were not being a resident of Mexico City or having plans to leave the area in the following 5 years, having a psychiatric disorder, daily consumption of alcoholic beverages, addiction to illegal drugs, continuous use of prescription drugs, diagnosis of high-risk pregnancy, preeclampsia, renal or circulatory disease including hypertension, gestational diabetes, suffering from seizures that required medical treatment and being pregnant with more than 14 weeks of gestation. A total of 327 and 670 subjects were enrolled in the first and second cohorts respectively. However, due mainly to budget considerations, only 194 subjects of the 1^st ^cohort and 118 of the second cohort had valid measurements of blood and plasma Pb in the 1^st ^trimester of pregnancy and provided information on history of abortion, Of these 312 subjects, 105 women with no previous pregnancies were excluded from this analysis since they had not been in risk of suffering an abortion (see statistical analysis section). In conclusion, the final sample was comprised of 207 women; 130 and 77 from the first and second cohorts, respectively (Figure 1).

All subjects were informed in detail about the nature and aims of the research studies and received information on ways to minimize their exposure to Pb. Those who accepted to participate read and signed a letter of informed consent. The research protocols were approved by the Ethics Committee of the National Institute of Public Health of Mexico and the Institutional Review Board of the Harvard University School of Public Health.

### Data collection

#### Assessment of outcome and covariates

We collected information on reproductive history by interviewing the participants during their first visit to the Center for Environmental Health Research of the American British Cowdray (ABC) Hospital in Mexico City, Mexico, scheduled at 12 weeks (± 2 weeks) of pregnancy. Information on the number of lifetime spontaneous abortions was collected via the question: *How many miscarriages (losses of pregnancy) have you had?*. Information on other reproductive history variables, diet, anthropometry, schooling and possible exposure to Pb was also collected by interview. The interviews and anthropometry were performed by previously standardized personnel.

#### Blood and plasma lead measurement

Blood and plasma samples were collected during each visit of the subjects to the research center. Subjects were instructed to fast overnight prior to sample collection. Prior to venipuncture, each subject's arm was washed with ultrapure water and disinfected with reagent-grade alcohol. Three cm^3 ^of venous whole blood was collected with a butterfly catheter (19 gauge) into a low lead container (Vacutainer, B-D 367734; Becton-Dickinson, Franklin Lakes, NJ) for blood lead analysis, and 13 cm^3 ^of venous blood was then collected into a polyethylene tube containing 100 USP of sodium heparin (H-3393; Sigma Chemical Company, St. Louis, MO), processed and shipped to the trace metal facility at the University of California, Santa Cruz for measurement of whole blood lead and plasma lead using ultra-clean methods detailed elsewhere [16]. All samples were analyzed using Inductively Coupled Plasma Mass Spectrometry (ICP-MS, Thermo Finnigan, Bremen, Germany). Potential contamination by lead from hemolyzed red cells was assessed by measuring levels of plasma iron and free hemoglobin using methods previously described in detail [12,16]. Using these procedures, 14 samples were determined to be contaminated with Pb from hemolysis suffered during sample collection and excluded from further analyses.

Cord blood was obtained when the subjects gave birth and was analyzed for Pb concentrations using a graphite furnace atomic absorption spectrophotometer (Perkin-Elmer 2100, Wellesley, MA, USA) at the trace metal laboratory of the ABC hospital in Mexico City. All sample collections were performed by personnel trained and standardized for the collection of blood and plasma following previously reported ultra clean procedures [12,16].

#### Bone Pb measurement

Valid data on patellar and tibial bone Pb concentrations (excluding measurements with uncertainties greater than 15 or 10 ppb of Pb for patella and tibia respectively) were available in a subsample of 153 and 95 women respectively. The measurements were obtained within four weeks of delivery using a spot-source ^109^Cd K-XRF instrument constructed at Harvard University and installed in a research facility in the American British Cowdray Medical Center. Thirty minutes of measurements were performed on the patella and midtibial shaft of each leg, representing trabecular and cortical bone, respectively.

### Statistical Analysis

Descriptive statistics were obtained and simple non-parametric statistical comparisons (Mann-Whitney test) were performed between those with no history of miscarriage vs. those with one or more events. A simple linear regression model of the plasma-to-blood Pb ratio was fitted to assess the relationship between this marker and the number of lifetime abortions; for this model, log_e _transformation of the plasma/blood Pb ratio was used to achieve a normal distribution of the residuals. Adjusted Incidence Rate Ratios (IRR) were estimated by fitting a Poisson regression model. Since women are only at risk of suffering miscarriage during pregnancy and consequently those with a larger number of pregnancies were at a larger risk of suffering a miscarriage, we defined the Incidence Rate of miscarriages for subject *i *as:

*r*_*i *_= *m*_*i*_/*p*_*i*_

where *r*_*i *_is the Incidence Rate of miscarriage for the i-th subject (i = 1, ..., n), *m*_*i *_is the number of miscarriages for the i-th subject and *p*_*i *_is the number of previous pregnancies. The current pregnancy was not considered as time of exposure since being currently pregnant was an inclusion criterion for all studied women. This means that subjects must not have an abortion in order to reach the first trimester of pregnancy and be included in the sample and thus none of them were "at risk" of having a miscarriage. It follows that the Incidence Rate of miscarriage was not defined for those with no previous pregnancies and therefore these women were not included in the analysis. Based on these considerations, we fitted the following Poisson regression model of the Incidence Rate of miscarriage:

*r*_*i *_= exp{*β*_0 _+ *β*_1_*RATIO*_*i *_+ *β*_2_*YSCH*_*i *_+ *β*_3_*AGE*_*i*_}

Where *RATIO*_*i *_is the plasma/blood Pb ratio, *YSCH*_*i *_are the number of years in school and *AGE*_*i *_is the age of the i-th subject. A second Poisson model features tertiles of the Plasma/Blood Pb ratio as an explanatory variable. Alternative models were fitted where whole blood Pb, plasma Pb and bone Pb were included as explanatory variables instead of the Plasma/Blood Pb ratio, these latter models included standardized independent variables, and the estimated IRRs are per one standard-deviation change in the explanatory variable. A secondary analysis was carried out to assess the possible correlation between cord blood lead levels during pregnancy and the plasma/blood Pb ratio; this hypothesis was tested by means of Cuzick's non-parametric test for trend.

#### Procedures for testing the Poisson assumption

In order to verify the adequacy of fitting Poisson regression models to spontaneous abortion incidence, we verified the assumption that spontaneous abortion data are Poisson distributed. We tested this assumption using three different approaches. The first two are based on the variance-to-mean ratio and the third one is based on the properties of the probability-generating function. Besides the *χ*^2^-test, one of the most common tests for verifying the Poisson assumption is the variance test (VT), also known as the index of dispersion test [17]:

VT=(n−1)S2X¯
 MathType@MTEF@5@5@+=feaafiart1ev1aaatCvAUfKttLearuWrP9MDH5MBPbIqV92AaeXatLxBI9gBaebbnrfifHhDYfgasaacH8akY=wiFfYdH8Gipec8Eeeu0xXdbba9frFj0=OqFfea0dXdd9vqai=hGuQ8kuc9pgc9s8qqaq=dirpe0xb9q8qiLsFr0=vr0=vr0dc8meaabaqaciaacaGaaeqabaqabeGadaaakeaacqWGwbGvcqWGubavcqGH9aqpcqGGOaakcqWGUbGBcqGHsislcqaIXaqmcqGGPaqkdaWcaaqaaiabdofatnaaCaaaleqabaGaeGOmaidaaaGcbaWaa0aaaeaacqWGybawaaaaaaaa@38BE@

It is known that *VT/(n-1) *is asymptotically a *χ*^2^-distribution with 1 degree of freedom. The power of the VT depends on the distribution under the alternative hypothesis. For spontaneous abortion data: *VT/(n-1) =*1.0676, *χ*^2^_*0.5,1 *_= *3.841*, *p *= *0.3014*. The asymptotically locally most powerful test (among all locally unbiased tests for testing the Poisson assumption against a mixed Poisson distribution) is given by Böhning [18], and Karlis & Xekalaki [19]:

O2=n−12(S2X¯−1)
 MathType@MTEF@5@5@+=feaafiart1ev1aaatCvAUfKttLearuWrP9MDH5MBPbIqV92AaeXatLxBI9gBaebbnrfifHhDYfgasaacH8akY=wiFfYdH8Gipec8Eeeu0xXdbba9frFj0=OqFfea0dXdd9vqai=hGuQ8kuc9pgc9s8qqaq=dirpe0xb9q8qiLsFr0=vr0=vr0dc8meaabaqaciaacaGaaeqabaqabeGadaaakeaacqWGpbWtdaWgaaWcbaGaeGOmaidabeaakiabg2da9maakaaabaWaaSaaaeaacqWGUbGBcqGHsislcqaIXaqmaeaacqaIYaGmaaaaleqaaOWaaeWaaeaadaWcaaqaaiabdofatnaaCaaaleqabaGaeGOmaidaaaGcbaWaa0aaaeaacqWGybawaaaaaiabgkHiTiabigdaXaGaayjkaiaawMcaaaaa@3B82@

This statistic is asymptotically standard normal distributed. For spontaneous abortion data: *O*_*2 *_= 0.6063, *Z*_*0.5 *_= *1.96*, *p *= *0.4925*. The probability generating function *φ(t) *of a Poisson distribution with mean *λ*, satisfies the relationship [20]:

∂(log⁡φ(t))∂t=λ
 MathType@MTEF@5@5@+=feaafiart1ev1aaatCvAUfKttLearuWrP9MDH5MBPbIqV92AaeXatLxBI9gBaebbnrfifHhDYfgasaacH8akY=wiFfYdH8Gipec8Eeeu0xXdbba9frFj0=OqFfea0dXdd9vqai=hGuQ8kuc9pgc9s8qqaq=dirpe0xb9q8qiLsFr0=vr0=vr0dc8meaabaqaciaacaGaaeqabaqabeGadaaakeaadaWcaaqaaGGaciab=jGi2kabcIcaOiGbcYgaSjabc+gaVjabcEgaNjab=z8aMjabcIcaOiabdsha0jabcMcaPiabcMcaPaqaaiab=jGi2kabdsha0baacqGH9aqpcqWF7oaBaaa@3E53@

that is, the function log(*φ(t)*) is a straight line with slope equal to the mean value *λ*.

Let *Y*_*n*_*(t) *denote the sample version of log(*φ(t)*):

Yn(t)=log⁡(φn(t)):=log⁡(1n∑i=1ntxi)t∈(0,1)
 MathType@MTEF@5@5@+=feaafiart1ev1aaatCvAUfKttLearuWrP9MDH5MBPbIqV92AaeXatLxBI9gBaebbnrfifHhDYfgasaacH8akY=wiFfYdH8Gipec8Eeeu0xXdbba9frFj0=OqFfea0dXdd9vqai=hGuQ8kuc9pgc9s8qqaq=dirpe0xb9q8qiLsFr0=vr0=vr0dc8meaabaqaciaacaGaaeqabaqabeGadaaakeaafaqabeqacaaabaGaemywaK1aaSbaaSqaaiabd6gaUbqabaGccqGGOaakcqWG0baDcqGGPaqkcqGH9aqpcyGGSbaBcqGGVbWBcqGGNbWzcqGGOaakiiGacqWFgpGzdaWgaaWcbaGaemOBa4gabeaakiabcIcaOiabdsha0jabcMcaPiabcMcaPiabcQda6iabg2da9iGbcYgaSjabc+gaVjabcEgaNnaabmaabaWaaSaaaeaacqaIXaqmaeaacqWGUbGBaaWaaabCaeaacqWG0baDdaahaaWcbeqaaiabdIha4naaBaaameaacqWGPbqAaeqaaaaaaSqaaiabdMgaPjabg2da9iabigdaXaqaaiabd6gaUbqdcqGHris5aaGccaGLOaGaayzkaaaabaGaemiDaqNaeyicI4SaeiikaGIaeGimaaJaeiilaWIaeGymaeJaeiykaKcaaaaa@5D1A@

Therefore, under the Poisson hypothesis and when *n *is large, the shape of *Y*_*n*_*(t) *is nearly a straight line. For the spontaneous abortion data,*φ*_*n*_*(t) *simplifies as :

φn(t)=1207∑i=1207txi=1207(136+59t+9t2+2t3+t4)t∈(0,1)
 MathType@MTEF@5@5@+=feaafiart1ev1aaatCvAUfKttLearuWrP9MDH5MBPbIqV92AaeXatLxBI9gBaebbnrfifHhDYfgasaacH8akY=wiFfYdH8Gipec8Eeeu0xXdbba9frFj0=OqFfea0dXdd9vqai=hGuQ8kuc9pgc9s8qqaq=dirpe0xb9q8qiLsFr0=vr0=vr0dc8meaabaqaciaacaGaaeqabaqabeGadaaakeaafaqabeqacaaabaacciGae8NXdy2aaSbaaSqaaiabd6gaUbqabaGccqGGOaakcqWG0baDcqGGPaqkcqGH9aqpdaWcaaqaaiabigdaXaqaaiabikdaYiabicdaWiabiEda3aaadaaeWbqaaiabdsha0naaCaaaleqabaGaemiEaG3aaSbaaWqaaiabdMgaPbqabaaaaaWcbaGaemyAaKMaeyypa0JaeGymaedabaGaeGOmaiJaeGimaaJaeG4naCdaniabggHiLdGccqGH9aqpdaWcaaqaaiabigdaXaqaaiabikdaYiabicdaWiabiEda3aaadaqadaqaaiabigdaXiabiodaZiabiAda2iabgUcaRiabiwda1iabiMda5iabdsha0jabgUcaRiabiMda5iabdsha0naaCaaaleqabaGaeGOmaidaaOGaey4kaSIaeGOmaiJaemiDaq3aaWbaaSqabeaacqaIZaWmaaGccqGHRaWkcqWG0baDdaahaaWcbeqaaiabisda0aaaaOGaayjkaiaawMcaaaqaaiabdsha0jabgIGiolabcIcaOiabicdaWiabcYcaSiabigdaXiabcMcaPaaaaaa@6696@

The behavior of *Y*_*n*_*(t)*, based on one hundred randomly chosen values of *t *within the interval (0,1), was practically a straight line. The slope of the fitting a regression model for these points was 0.4191 practically equal to the mean of the number of miscarriages. All three tests indicate a high agreement with the Poisson assumption.

## Results

Descriptive statistics are presented in table 1. Age ranged from 17 to 43 years, the number of years in school varied between zero and 18, and the number of previous pregnancies ranged between 1 and 5. Average plasma/blood Pb ratio was 0.22 percent (range: 0.01 to 0.99 percent), history of abortions ranged from 0 to 4 (mean: 0.42), 34.3 percent of the women reported having experienced at least one miscarriage and 4.3 percent reported having had two or more. Women who reported having had at least one abortion were in average slightly less educated (9.49 vs. 9.22 years in school) and had a higher average number of pregnancies (2.16 vs. 1.38) than women with no previous abortions. Women with a history of miscarriage had a comparatively higher mean plasma/blood Pb ratio relative to those with no history.

**Table 1:** Characteristics of participants. Mexico City, Mexico, 1997–2004

	**All subjects (n = 207)**		**No miscarriages (n = 136)**		**≥ 1 miscarriage (n = 71)**		
**Variable**	**Mean**	**SD†**	**Mean**	**SD**	**Mean**	**SD**	p*

Age (years)	27.76	5.33	27.57	4.88	28.11	6.13	0.60
Years in school	9.4	4.04	9.49	3.82	9.23	4.45	0.90
Pregnancies	1.65	0.90	1.38	0.64	2.17	1.08	<0.01
Miscarriages	0.42	0.67	--	--	1.23	0.57	--
Blood Pb (μg/L)	62.4	44.82	64.73	49.46	57.95	34.09	0.37
Plasma Pb (μg/L)	0.14	0.13	0.13	0.13	0.14	0.13	0.15
Plasma Pb/Blood Pb ratio (%)	0.22	0.14	0.21	0.13	0.25	0.17	0.02

* Mann-Whitney test

† SD: standard deviation

**Figure 2:**
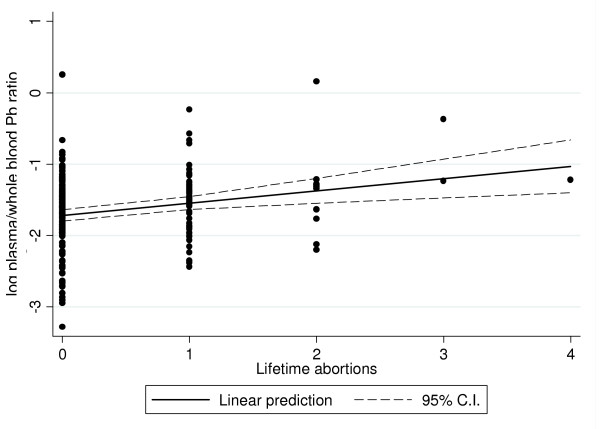
**Regression of the plasma/blood Pb ratio on the number of miscarriages. Mexico City, Mexico, 1997–2004**. Simple ordinary least squares regression model of the plasma/blood Pb ratio of lead as a function of the lifetime number of spontaneous abortions.

There appears to be a linear trend of the plasma/blood Pb ratio according to the reported number of lifetime abortions, which is seen in figure 2. According to this linear model, women with one additional abortion have an 18 percent greater plasma-to-blood Pb ratio (*p *< 0.01), this relationship held even after removing the three subjects with more than 2 miscarriages (16 percent greater plasma/blood Pb per additional abortion, *p *= 0.02). Additionally, adjusting for the number of pregnancies did not have a noticeable impact in the coefficient estimate (19 percent greater plasma/blood Pb per additional abortion, *p *< 0.01).

Women in the upper tertile of the plasma/blood Pb ratio during the 1^st ^trimester of pregnancy had almost twice the incidence rate of reported spontaneous abortions compared to women in the lower tertile, (IRR = 1.90, *p *= 0.02), after adjustment for covariates, women on the second tertile showed a 16 percent greater Incidence Rate of miscarriage although it was not statistically significant (*p *= 0.61). When the plasma/blood Pb ratio was analyzed as a continuous variable, we documented that an increment of 0.1 percentage points in the plasma/blood Pb ratio is associated with a 12 percent greater incidence rate of abortion (IRR = 1.12, *p *= 0.02); both models are presented in table 2. These models included schooling as a proxy of socioeconomic status, however, this variable was not found to be a determinant of the incidence of abortions and did not seem to exert a confounding effect on the PPb/BPb effect estimate (IRR for continuous PPb/BPb ratio without adjustment for schooling was 1.121, p = 0.018). When we tested models including whole blood, plasma or bone Pb concentrations as predictor variables instead of the plasma-to-blood Pb ratios, no significant association with history of spontaneous abortion was found; a comparison of the effect estimates of the different Pb biomarkers is presented in table 3. We also tested for an interaction between bone Pb concentrations and the plasma/blood Pb ratio; according to our hypothesis, we could expect the effect of bone Pb on history of miscarriage to be greater on those women with higher ratios. We found the interaction term to be in the expected direction in the patella model (interaction IRR = 1.14, *p *= 0.21), but no significant interaction was observed in the tibia model (interaction IRR = 1.01, p = 0.99).

**Table 2:** Poisson regression models of history of abortion. Mexico City, Mexico, 1997–2004

**Variable (units)**	**Continuous model**	**Quantiles model**
	IRR*	IRR*

Plasmatic Pb fraction^†^	1.123	
	[0.017]	
Plasmatic Pb fraction tertiles		
1st		1.00
		--
2nd		1.161
		[0.612]
3rd		1.903
		[0.015]
Age (years)	0.985	0.984
	[0.477]	[0.432]
Schooling (years)	1.025	1.021
	[0.386]	[0.455]

Poisson multiple regression models of the rate of spontaneous abortions featuring continuous (left column) or categorized (right column) plasma/blood Pb ratio as predictor variables, both models are adjusted for age and schooling

* Incidence Rate Ratio, p-values in brackets

† IRR for a 0.1 percentage point increase in the plasmatic fraction of Pb

**Table 3:** Incidence Rate Ratios of miscarriage comparing different biomarkers of lead exposure. Mexico City, Mexico, 1997–2004

**Biomarker**	IRR*	***p***
Plasma Pb	1.12	0.22
Blood Pb	0.93	0.56
Plasma/Blood Pb ratio	1.18	0.02
Patella Pb^†^	1.15	0.39
Tibia Pb^‡^	1.07	0.56

Comparison of incidence of spontaneous abortion rate ratios per increase of one standard deviation of several biomarkers of lead exposure. Note: Each line features an effect estimate obtained by separate Poisson regression models.

* Incidence Rate Ratio, adjusted for age and schooling.

^† ^n = 95

^‡ ^n = 153

Cord blood lead levels were measured in a sample of 206 subjects, of whom 113 mothers had valid plasma Pb measurements. We performed a secondary analysis on these mother-child pairs, regardless of them being first pregnant, to assess if plasma to blood Pb ratios were correlated to cord blood lead levels. Cord blood was apparently correlated with the plasma/whole blood Pb ratio; children whose mothers were in the upper tertile of the plasma/blood Pb ratio in the first trimester of pregnancy had a mean cord blood Pb of 4.61 μg/dL, compared with 5.03 and 6.36 μg/dL corresponding to the 2^nd ^and 3^rd ^tertile (*p*-value for trend = 0.06).

## Discussion

The results obtained by this study suggest that the plasma/blood Pb ratio could be viewed as a marker of susceptibility for lead toxicity. Our findings are consistent with studies relating blood lead and risk of spontaneous abortion [1]; a high plasma/blood Pb ratio implies more circulating lead is free to cross the placenta at a given blood lead level. This is consistent with our finding of a correlation between plasma/blood Pb ratios and cord blood Pb concentration at birth. If inter-individual factors such as polymorphisms of the ALAD gene determine the plasma/blood lead ratio [5], then some women, if exposed to lead during pregnancy, would be at increased risk of fetal lead exposure. The rationale for the latter idea is that if a pregnant woman is acutely exposed to lead from, for example, eating from a lead-glazed pot, her erithrocytic lead binding capacity will determine how much lead remains free in plasma and potentially reaches the fetus. This hypothesis is consistent with our observations: fetuses of women who tend to have a higher plasma/blood Pb ratio would be more susceptible to lead exposure and therefore these women would have a history of spontaneous abortions in previous pregnancies. In accordance with our hypothesis, a lack of a strong association between plasma Pb concentrations and history of previous occurrence of abortions was indeed observed in our sample as presented in table 3. The relatively large effect estimates of bone Pb are worthwhile mentioning, since it would be expected that women with a high lead burden are at elevated risk of spontaneous abortion. Furthermore, the positive patella Pb-ratio interaction term estimate suggests that the association between bone Pb and history of abortion is greater in those women with higher plasma/blood Pb ratios, which is in accordance to the hypothesis presented here. However, the small sample size of women with valid bone Pb measurements in our study prevented us from further exploring this hypothesis and reaching any definitive conclusion.

Our study has several limitations worth discussing. Given we are trying to find an association between a biomarker measured in the present time to events occurred years before, no causal relationship can be established. However, under the assumption that certain factors that determine the percentage of free circulating lead in humans remain constant between pregnancies, these findings would imply that women who have a lower red cell binding capacity for circulating lead may be at increased risk of suffering from spontaneous abortion. A second limitation relies on the fact that history of miscarriage was self-reported. This has the limitation that some women may suffer a spontaneous abortion without knowing that they were pregnant, while other women desirous of being pregnant may also report a spontaneous abortion without actually being pregnant. Nevertheless, this imprecision in the number of reported miscarriages is unlikely to be correlated to the plasma/blood Pb ratio and therefore our estimates are unlikely to be biased. This random measurement error of the outcome, however, may be reflected in imprecise effect estimates.

A different issue is that our sample constitutes a relatively small fraction of the cohorts which it arose from. If somehow the probability of being selected into the study sample was related to both plasma/blood Pb ratios and the number of miscarriages, selection bias could ensue. In order to test if being selected into the final sample depended on observed characteristics of the subjects, especially those related to the outcome and the exposure, we fitted the following empirical model by means of logistic regression:

ln(*p*_*i*_/1 - *p*_*i*_) = *α*_0 _+ *α*_1_*A*_*i *_+ *α*_2_*BPb*_*i *_+ *α*_3_*C*_*i *_+ X'_i_β

Where *p*_*j *_is a the probability of participation in the study, *A*_*i *_denotes the number of spontaneous abortions for the i-th subject; *BPb*_*i *_is the blood lead concentration of the subject, *C*_*i *_is an indicator variable for the subject's cohort and **X'**_*i *_is a vector of individual characteristics such as age, number of years in school, number of pregnancies, weight and height (Table 4). Using this approach, we found no evidence of selection bias; neither of the explored variables was associated to the probability of participating in the study except for the cohort indicator. The reason for the latter is the greater percentage of subjects in the first cohort who had a plasma Pb measurement and were therefore included in the study sample.

**Table 4:** Logistic regression model of participation in the study. Mexico City, Mexico, 1997–2004

**Variable (units)**	Odds Ratio*	***p***
Number of abortions	1.15	0.46
Number of pregnancies	1.10	0.53
Age (years)	0.99	0.55
Schooling (years)	1.00	0.97
Weight (kg)	1.02	0.17
Height (cm)	0.98	0.36
Blood Pb (μg/L)	1.00	0.36
Cohort (0 = 1st, 1 = 2nd)	0.07	<0.01

* Relative odds of participation in the study.

Another limitation was our inability to address the issue of lead and risk of spontaneous abortion in current pregnancies since women were recruited past the point at which spontaneous abortion usually occurs, and as a consequence the incidence of abortion in the study cohort was very small (1.3 percent), trying to estimate the effect of lead on the outcome of this particular pregnancy will be likely biased since women who miscarried before week 10 (including events possibly due to lead) had zero probability of being included in this sample. Abortions in past pregnancies could have occurred at any gestational age and thus our estimates would not be affected by this issue. However, there is a possibility that, if our hypothesis is true, women with very high plasma/blood Pb ratios could suffer from frequent miscarriages. For such women, the probability to reach 10 weeks of gestation and become eligible for this study would be low. If this were the case, it is likely that the true effect of the plasma/blood Pb ratio on the risk of miscarriage is underestimated by our study. This possibility is supported by the data as the mean of the plasma/blood Pb ratio in our study sample (0.22 percent) was lower than that found in a sample of comparable non-pregnant Mexico City residents (0.31 percent) using the same methodology for plasma and blood Pb measurement [12].

Finally, we should note that social class could be viewed as an important potential confounder. Since both cohorts had the limitation of not registering a socioeconomic index, the models we presented adjust for schooling as a proxy of socioeconomic status. The fact that it did not seem to act as a confounder is consistent with the idea that plasma/blood Pb ratios are governed by biological individual characteristics rather than social factors. This is reflected on the finding that schooling was not correlated with the plasma/blood Pb ratio in our study sample (Spearman's ρ = -0.03, p = 0.67).

## Conclusion

Our results constitute evidence that the history of spontaneous abortion is related to the plasma/blood Pb ratio, which could be due to a greater availability of placental barrier-crossing Pb for a given blood Pb concentration in some women. Nevertheless, specifically designed longitudinal studies on this issue will be necessary to verify these hypotheses. Assessing the influence of genetic polymorphisms of lead binding proteins on the probability of suffering from miscarriage or other reproductive outcomes will be very important to identify groups particularly susceptible to the effects of lead exposure during pregnancy.

## Competing interests

The author(s) declare that they have no competing interests.

## Authors' contributions

HLF formulated the hypothesis, carried out part of the statistical analysis, prepared the background, results and discussion sections, as well as part of the methods section. MMTR collaborated in the statistical analysis and interpretation of results, MHA and HH designed the original studies whence the data for this study originated, and contributed to the discussion of the findings. MSG constructed the datasets and carried out part of the statistical analysis and the literature review. AMG was the field supervisor and reviewed the manuscript, ROW contributed to the discussion and interpretation of results. DS implemented the technique and supervised the procedures for plasma and whole-blood Pb measurements; he also contributed to the discussion of the findings. BTV designed and validated the statistical analysis approach and wrote part of the methods section. All authors read and approved the final version of the manuscript.

## Pre-publication history

The pre-publication history for this paper can be accessed here:



## References

[B1] Borja-Aburto VH, Hertz-Picciotto I, Rojas-Lopez M, Farias P, Rios C, Blanco J (1999). Blood lead levels measured prospectively and risk of spontaneous abortion. Am J Epidemiol.

[B2] Tang N, Zhu ZQ (2003). Adverse reproductive effects in female workers of lead battery plants. Int J Occup Med Environ Health.

[B3] Cengiz B, Soylemez F, Ozturk E, Cavdar AO (2004). Serum zinc, selenium, copper, and lead levels in women with second-trimester induced abortion resulting from neural tube defects: a preliminary study. Biol Trace Elem Res.

[B4] Hertz-Picciotto I (2000). The evidence that lead increases the risk for spontaneous abortion. Am J Ind Med.

[B5] Hernandez-Avila M, Smith D, Meneses F, Sanin LH, Hu H (1998). The influence of bone and blood lead on plasma lead levels in environmentally exposed adults. Environ Health Perspect.

[B6] Chuang HY, Schwartz J, Gonzales-Cossio T, Lugo MC, Palazuelos E, Aro A, Hu H, Hernandez-Avila M (2001). Interrelations of lead levels in bone, venous blood, and umbilical cord blood with exogenous lead exposure through maternal plasma lead in peripartum women. Environ Health Perspect.

[B7] Bergdahl IA, Vahter M, Counter SA, Schutz A, Buchanan LH, Ortega F, Laurell G, Skerfving S (1999). Lead in plasma and whole blood from lead-exposed children. Environ Res.

[B8] Hu H, Téllez-Rojo MM, Bellinger DC, Smith D, Ettinger AS, Lamadrid-Figueroa H, Schwartz J, Schnaas L, Mercado-Garcia A, Hernandez-Avila M (2006). Fetal Lead Exposure at Each Stage of Pregnancy as a Predictor of Infant Mental Development. Environmental Health Perspectives. Environ Health Perspect.

[B9] Faikoglu R, Savan K, Utku C, Takar N, Zebitay AG (2006). Significance of maternal plasma lead level in early pregnancy loss. J Environ Sci Health A Tox Hazard Subst Environ Eng.

[B10] Bergdahl IA, Gerhardsson L, Liljelind IE, Nilsson L, Skerfving S (2006). Plasma-lead concentration: investigations into its usefulness for biological monitoring of occupational lead exposure. Am J Ind Med.

[B11] Lamadrid-Figueroa H, Tellez-Rojo MM, Hernandez-Cadena L, Mercado-Garcia A, Smith D, Solano-Gonzalez M, Hernandez-Avila M, Hu H (2006). Biological markers of fetal lead exposure at each stage of pregnancy. J Toxicol Environ Health A.

[B12] Smith D, Hernandez-Avila M, Tellez-Rojo MM, Mercado A, Hu H (2002). The relationship between lead in plasma and whole blood in women. Environ Health Perspect.

[B13] Bergdahl IA, Grubb A, Schutz A, Desnick RJ, Wetmur JG, Sassa S, Skerfving S (1997). Lead binding to delta-aminolevulinic acid dehydratase (ALAD) in human erythrocytes. Pharmacol Toxicol.

[B14] Bergdahl IA, Sheveleva M, Schütz A, Artamonova VG, Skerfving S (1998). Plasma and blood lead in humans: capacity-limited binding to delta-aminolevulinic acid dehydratase and other lead-binding components. Toxicol Sci.

[B15] Montenegro MF, Barbosa F, Sandrim VC, Gerlach RF, Tanus-Santos JE (2006). A polymorphism in the delta-aminolevulinic acid dehydratase gene modifies plasma/whole blood lead ratio. Arch Toxicol.

[B16] Smith D, Ilustre R, Osterloh J (1998). Methodological considerations for the accurate determination of lead in human plasma and serum. Am J Ind Health.

[B17] Cochran W (1954). Some methods of strengthening the X^2 ^goodness of fit test. Biometrics.

[B18] Böhning D (1994). A note on a test for Poisson overdispersion. Biometrika.

[B19] Karlis D, Xekalaki E (2000). A simulation comparison of several procedures for testing the Poisson assumption. The Statistician.

[B20] Nakamura M, Perez-Abreu V (1993). Use of an empirical probability generating function for testing a Poisson model. Can J Statist.

